# Crystalglobulin-associated nephropathy presenting as MGRS in a case of monoclonal B-cell lymphocytosis: a case report

**DOI:** 10.1186/s12882-020-01818-5

**Published:** 2020-05-18

**Authors:** Rajib K. Gupta, Lois J. Arend, Anupama BK, Sriram Narsipur, Ramya Bhargava

**Affiliations:** 1 Department of Pathology, SUNY Upstate Medical University, 750 E Adams Street, Syracuse, NY 13210 USA; 2 Department of Pathology, Johns Hopkins University School of Medicine, Baltimore, MD USA; 3 Department of Medicine, SUNY Upstate Medical University, Syracuse, NY USA; 4 Department of Medicine and Nephrology, SUNY Upstate Medical University, Syracuse, NY USA; 5 Department of Nephrology, SUNY Upstate Medical University, Syracuse, NY USA

**Keywords:** Crystalglobulin-associated nephropathy, Monoclonal B-cell lymphocytosis, Monoclonal gammopathy of renal significance

## Abstract

**Background:**

Crystalglobulin-associated nephropathy (CAN), a rare subtype of monoclonal gammopathy, usually associated with multiple myeloma and occasionally monoclonal gammopathy of uncertain significance (MGUS), is characterized by occluding monoclonal pseudothrombi within renal glomerular capillaries and/or interstitial arterioles. Ultrastructurally, these pseudothrombi are unique for having a crystalline substructure. We describe a case of an adult patient with monoclonal B-cell lymphocytosis (MBL) and acute renal failure whose kidney biopsy revealed a rare diagnosis of CAN.

**Case presentation:**

A 63-year old male presented with a 2-month history of edema, arthralgia and malaise. He had acute kidney injury with hematoproteinuria on urine analysis. Serum and urine protein electrophoresis were both negative. A renal biopsy however revealed features of CAN. Organomegaly, bone pain and lymphadenopathy were absent. A repeat serum electrophoresis was positive for IgA kappa and a free light chain assay showed elevated free kappa light chains. Flow cytometry done subsequently revealed a diagnosis of MBL, chronic lymphocytic leukemia (CLL) type.

**Conclusion:**

CAN in association with MBL/CLL has not been previously described in literature, and our case highlights yet another instance of monoclonal gammopathy of renal significance (MGRS) where a small B-cell clone resulted in extensive renal pathology without systemic manifestations.

## Background

Crystalglobulin-associated nephropathy (CAN) is a monoclonal immunoglobulin light chain-related rare renal disease characterized by large extracellular crystals of paraproteins presenting as occluding thrombi (pseudothrombi) within glomerular capillaries and/or renal interstitial arterioles [1, 2]. Sometimes CAN is associated with a circulating monoclonal protein with cryoglobulin-like properties resulting in small vessel occlusion and inflammation in the systemic microvasculature, when it is known as crystalglobulinemia or cryocrystalglobulinemia [1]. In published literature, CAN has been described mainly in association with frank multiple myeloma (MM) or monoclonal gammopathy of undetermined significance (MGUS) [1]. We describe a rare case report of a patient with a small clonal B-cell population (also known as monoclonal B-cell lymphocytosis or MBL) who developed acute renal failure from CAN requiring long term hemodialysis. CAN presenting as a monoclonal gammopathy of renal significance (MGRS) in a MBL patient has not been described previously in literature.

## Case presentation

**Fig. 1:**
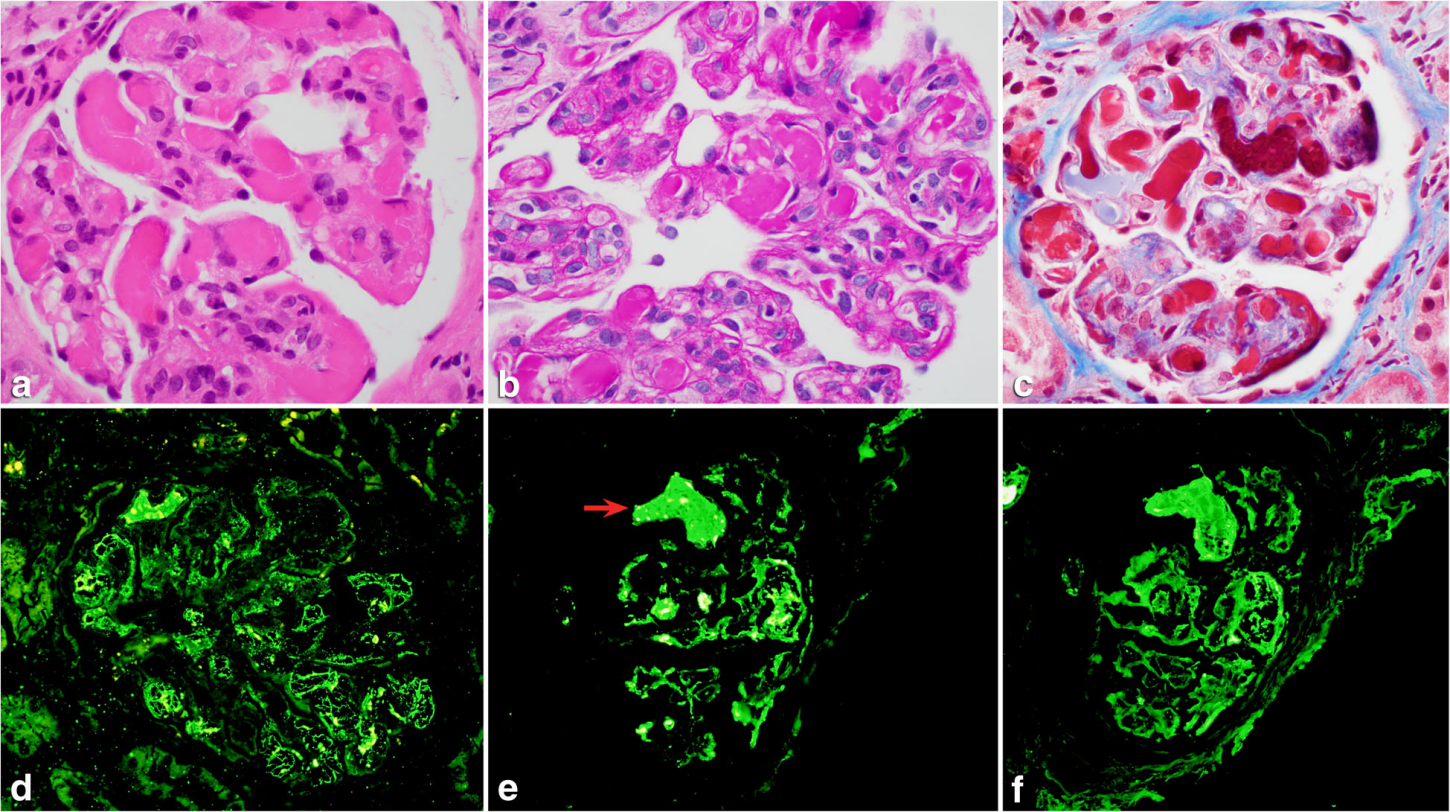
Light and immunofluorescence microscopy images: **a**. H&E section showing a representative glomerulus with capillary loops obstructed by pseudothrombi, X 600; **b-c**. PAS (**b**) and Masson trichrome (**c**) stained sections showing segmental to global occlusion of glomerular capillaries by pseudothrombi, X 600. The glomerulus in **b** also shows segmental double contours, and has a membranoproliferative appearance; **d**-**f** Immunofluorescence staining for IgA (**d**), kappa (**e**), and lambda (**f**) showing staining of the pseudothrombi for IgA, kappa, and lambda, with similar intensity. Within some pseudothrombi are intensely stained foci that are more numerous for IgA and kappa than lambda (arrow in image **e**)

**Fig. 2:**
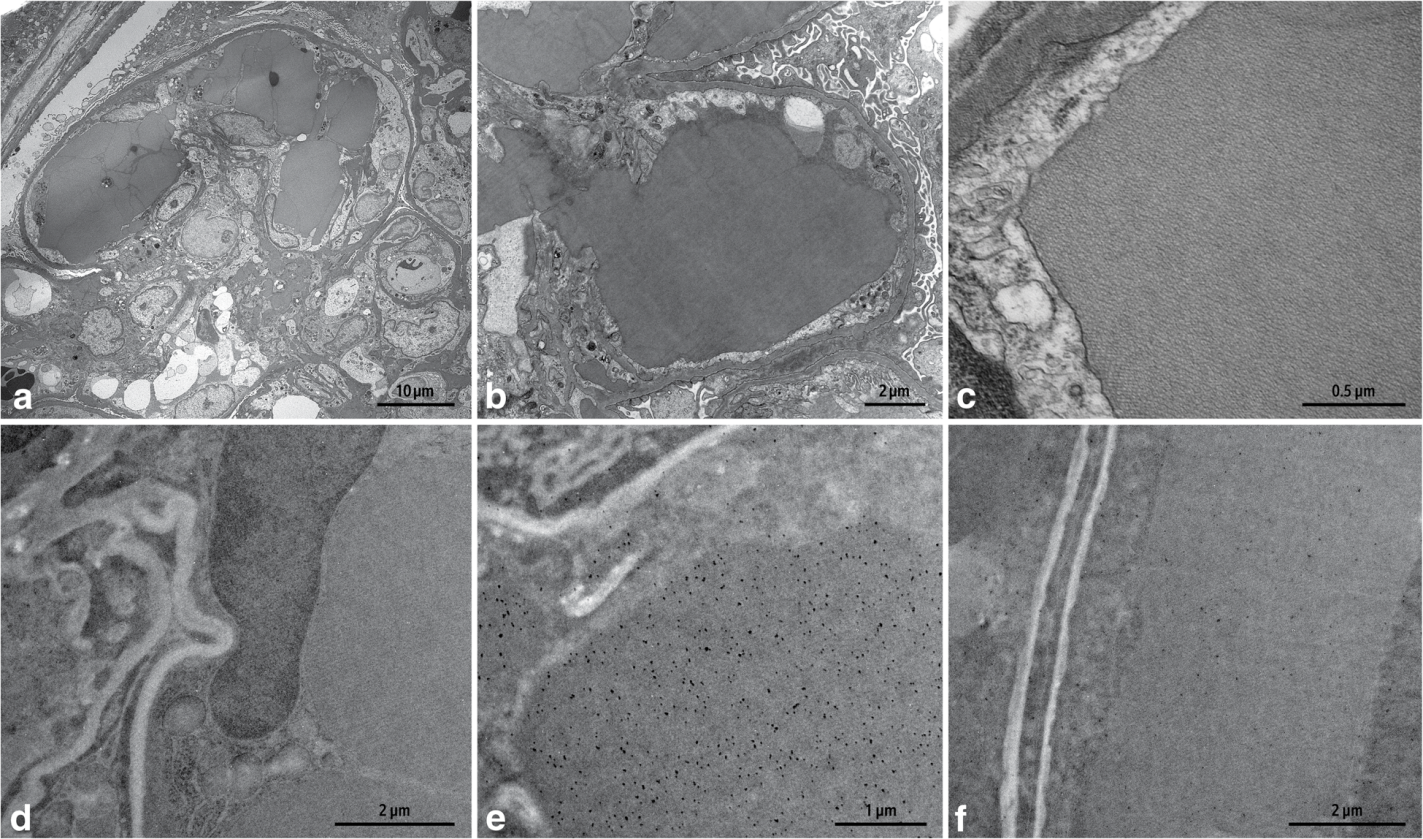
**a**-**c**. Electron microscopy images: **a***. * Glomerulus with adjacent capillary loops lined by swollen endothelial cells and occluded by several electron-dense crystals with sharp edges, magnification X 1900, **b**. Glomerular capillary loop with an electron-dense occluding crystal, magnification X 6800; this loop shows moderate podocyte foot process effacement, while few other loops (not shown) showed severe foot process effacement, **c**. Image showing a higher magnification of the same crystal in **b** revealing a lattice-like substructure, magnification X 49000. **d-f**. Immunogold electron microscopy images: **d**. Control without primary antibody (X 5000), **e**. Labeling of crystal with numerous kappa gold particles (X 8000), **f**. Crystal with scant lambda gold particles (X 5000). μm = microns

A 63-year-old male with hypertension and seizure disorder presented to the renal clinic with a blood pressure of 220/110 mmHg, headache and neck pain and was sent to the emergency department. His review of systems showed that he had a 2-month history of bilateral ankle pain, anasarca, progressive renal impairment, weakness and fatigue, associated with a history of loss of appetite for this period of time. A renal workup revealed nephrotic-range proteinuria and microscopic hematuria, with urinalysis showing red cells and protein. CBC showed a hemoglobin of 8.6 g/dL, white cell count of 7.7 K/uL and a platelet count of 146 K/uL. Blood chemistry showed sodium of 146 mmol/L. potassium of 4.5 mmol/L, bicarbonate of 25 mmol/L, chloride of 113 mmol/L, BUN of 61 mg/dL and a creatinine of 4.08 mg/dL. Serology was negative for antinuclear antibody, antineutrophil cytoplasmic antibodies, rheumatoid factor, anti-double stranded DNA antibody, lupus anticoagulant, cryoglobulins and viral markers (HBV, HCV and HIV). Complement levels were normal. Serum and urine protein electrophoresis were negative for a monoclonal protein. Although he had slightly low haptoglobin, his ADAMTS13 level was within normal limits. A renal core biopsy showed predominantly medulla and a small cortical sample, containing up to 12 glomeruli of which one glomerulus was globally sclerosed. All patent glomeruli showed segmental or global occlusion of capillary loops by PAS-positive eosinophilic pseudothrombi which were non-argyrophilic and stained magenta red on trichrome (Fig. 1a-c); some of the glomeruli had a membranoproliferative pattern associated with mild mesangial hypercellularity and segmental double contours (Fig. 1b, PAS stain). A single artery sampled showed moderate arteriosclerosis but no thrombi. The interstitium was unremarkable except for patchy fibrosis. On immunofluorescence microscopy (IF), the pseudothrombi stained for IgA, kappa and lambda (Fig. 1d-f) and were negative for the other immunoreactants; some pseudothrombi also showed intensely staining bright foci that were more numerous in kappa than in lambda (arrow). Electron microscopy (EM) of 2 glomeruli showed several capillary loops lined by swollen endothelial cells, with occluding intra-luminal electron-dense crystals; all the crystals showed a lattice-like substructure with parallel linear arrays. No conventional immune complex-type electron-dense deposits or fibrin tactoids were seen within the glomeruli (Fig. 2a-c). Crystals were also not seen within proximal tubular epithelial cells. Occasional loops showed mild subendothelial widening along with rare cell interposition. A diagnosis of crystalglobulin-associated nephropathy (CAN) with possible kappa restriction was favored. Paraffin-IF with pronase digestion could not be performed as no glomeruli remained in the paraffin block. Subsequent testing with immunogold EM showed preferential association of kappa particles for the crystals, confirming kappa restriction of the crystals (Fig. 2d-f). A repeat serum electrophoresis 3 months later showed IgA kappa and trace amount of IgG lambda while urinary protein electrophoresis remained negative; a free light chain assay showed a kappa/lambda ratio of 3.9. Bone marrow and peripheral blood flow cytometry at this time showed a small clonal B-cell population (0.19 K/uL) with kappa restriction and a chronic lymphocytic leukemia (CLL) phenotype, co-expressing CD5 and CD23 and being negative for CD10 and CD103. No organomegaly or lymphadenopathy was present in the patient. A diagnosis of CAN presenting as MGRS in the background of monoclonal B-cell lymphocytosis (MBL) was confirmed in the patient. Since the diagnosis, the patient has been dialysis-dependent. A hemato-oncology referral was sought in view of the patient’s MGRS finding but because of the low clonal B-cell count coupled with extreme frailty and recurrent episodes of C.difficile diarrhea in the patient, no specific hematologic treatment was decided to be initiated by the clinical team till the patient became clinically more stable.

## Discussion and conclusion

Monoclonal gammopathies causing renal lesions may be caused by an underlying hematologic disease which, in turn, may be frankly malignant conditions like symptomatic MM or a B-cell lymphoma or premalignant states like smoldering myeloma, MGUS and MBL (the B-cell counterpart of MGUS) [3]. Renal pathology in association with all premalignant hematologic diseases is now considered under the umbrella of MGRS [2, 3]. While MGRS in association with MGUS has been known for some time now, MGRS caused by nephrotoxic paraproteins released by premalignant small B-cell clones has only recently gained attention [3].

**Fig. 3:**
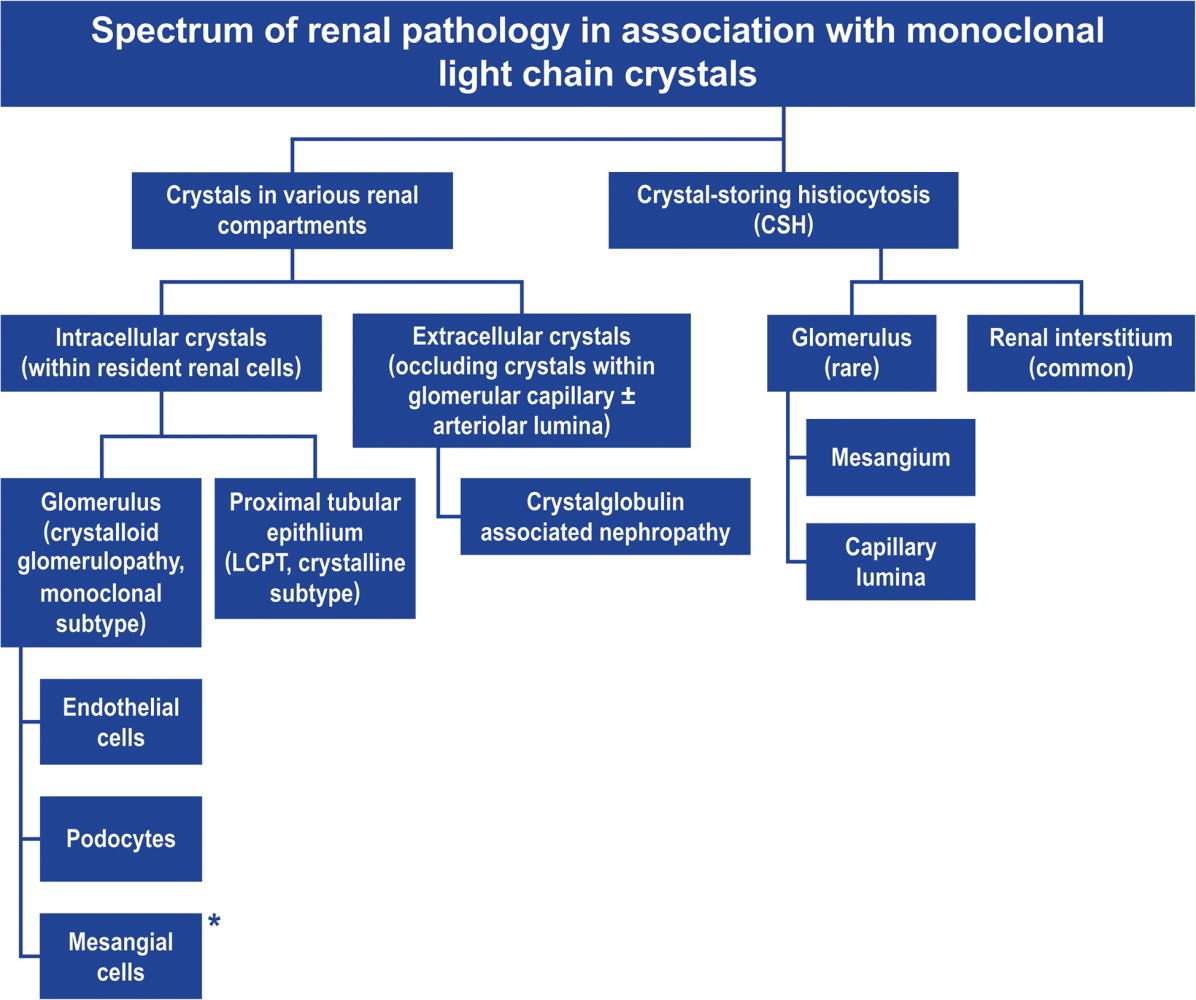
Flowchart depicting the spectrum of kidney involvement with monoclonal crystals. *Case involving pure monoclonal crystals within mesangial cells have not been reported yet in literature, although CSH within mesangial cells has been described, ref. [9]. This figure has been reproduced (with due permission, copyright Elsevier 2019) after slight modification of Fig. 5 in reference [8]

CAN is a rare paraprotein crystal-induced renal disease caused by glomerular intracapillary and renal arteriolar occlusion by crystals, the other subtypes of paraprotein crystal-induced nephropathies include - crystalline variant of light chain proximal tubulopathy (intracellular light chain crystals precipitating within the proximal tubular epithelial cells and often associated with Fanconi syndrome) [2, 4], monoclonal crystalloid glomerulopathy (intracellular light chain crystals within individual glomerular cell compartments like endothelial cells or podocytes) [5–7] and crystal-storing histiocytosis (light chain crystal-bearing histiocytes found either in interstitium, mesangium or glomerular capillary loops), as highlighted in Fig. 3 [8, 9]. Although there have been case reports of crystalglobulinemia since 1930s [10], it is only recently that a specific renal lesion in association with these extracellular circulating crystals has been delineated [1]. CAN has been described mostly in association with MM and MGUS [1, 11]. Our report describes a rare case of CAN associated with MBL (with CLL phenotype) presenting as MGRS in a patient with acute renal failure leading to hemodialysis. A B-cell clone under 5 K/uL falls under the MBL category as per latest WHO guidelines [12], and our patient’s clonal B-cell count was quite low, at 0.19 K/uL. Two large case series [13, 14] which have studied the spectrum of renal lesions associated with CLL/MBL have described various MGRS entities such as cryoglobulinemic glomerulonephritis (GN), immunotactoid GN, fibrillary GN, amyloidosis and light chain cast nephropathy, but CAN developing as a result of CLL/MBL has not been described previously. A 2014 series of 20 cases of non-Hodgkin lymphoma with biopsy-proven renal involvement describes a single case of CLL having light microscopy appearance similar to ours with intracapillary plugging by PAS-positive pseudothrombi which stained for IgM and kappa by IF but on EM, showed only granular electron-dense deposits occluding the capillary loops but without any substructure [15].

Histologically, the renal biopsy in CAN shows glomeruli with large intracapillary eosinophilic pseudothrombi with or without glomerular hypercellularity - while endocapillary hypercellularity has been variably described, mesangial hypercellularity (present in our case) has not been described in previous case reports [1, 11]. Segmental capillary wall double contours were described in one case [16]. The double contours seen in the glomeruli by light microscopy (and corroborated by subendothelial widening seen in some capillary loops on EM) were likely the result of endothelial injury from the patient’s severe hypertension or direct injury from the intraluminal occluding crystals, or both. Although classic features of thrombotic microangiopathy (TMA) such as fibrin thrombi or mucoid intimal thickening were not seen within the glomeruli or vasculature sampled (only one artery being present in the biopsy), the possibility of a monoclonal gammopathy-associated TMA cannot be completely excluded [17]. Frozen section immunofluorescence (IF) can be challenging to interpret in cases of CAN (as was in our case) and, where available, paraffin IF with pronase digestion, has been recommended as a more sensitive IF technique in such cases [18]. However, immunogold EM (IEM), can also serve as an alternative and equally (if not more) sensitive technique for confirming monoclonality of crystalline deposits by actually localizing kappa or lambda light chains within the crystals [8, 19, 20]. In our case, IEM proved indispensable in confirming the kappa restriction of the light chain intraluminal crystals. IEM for immunoglobulins was not attempted as it not standardized in the electron microscopy lab where the IEM procedure was done, and it has not been well described in literature [19, 20]. Patients with CAN (particularly those with cryocrystalglobulinemia) may develop symptoms like rash, arthralgia and peripheral neuropathy as usually seen in patients with cryoglobulinemic and rheumatic diseases [1], and even renal arterial thrombosis in rare instances [11]. Our patient had arthralgia but none of the other symptoms. Usually, kappa predominates as the restricted light chain that is overproduced in monoclonal crystal-related nephropathies [1, 11], although occasional lambda-predominant cases have been reported [16].

The histologic differential diagnoses for our case included – TMA associated with various etiologies like hemolytic-uremic syndrome, complement abnormality, malignant hypertension, autoimmune diseases (including antiphospholipid antibody syndrome) or solid malignancies, type 1 cryoglobulinemic glomerulonephritis secondary to kappa monoclonal disease or staphylococcal infection-related glomerulonephritis (SAGN) with cryoglobulinemic features. However, in none of these conditions are actual monoclonal crystals with lattice substructure found within capillary loops on EM as was seen in our case [21, 22]. Also in our case, the patient had no recent history of infection or pre-existing diabetes to support a diagnosis of SAGN.

The exact pathologic/biochemical basis of crystallization of monoclonal proteins and the reason for kappa predominance are not well understood. However researchers have suggested possible abnormal N-glycosylation of the light chain portion of monoclonal paraproteins, aided by stasis in microvasculature as the reason for crystal formation [23], while unusual hydrophobic residues at the variable (V) domain of the kappa light chain at position 30 or unusual amino acid substitutions in the V region have been cited as a reason why the kappa crystals are resistant to normal lysosomal degradation in the B- or plasma cell- microenvironment [24].

In conclusion, we describe a rare case of CAN in a patient with MBL (CLL subtype) and renal failure and without systemic symptoms, organomegaly, bone pain or lymphadenopathy. Our case highlights the importance of being aware of rare entities like CAN and also stresses the fact that even a small B-cell clone as in MBL should not be ignored [25] as it can sometimes lead to selective kidney dysfunction as MGRS. Our case also shows how IEM can be of immense help in clinching a diagnosis when standard immunofluorescence is equivocal or even false-negative [20]. Lastly, the case stresses the importance of doing repeat SPEP testing (when initial results are negative) and free light chain assay as a mandatory workup in any patient with suspicion of a monoclonal gammopathy, with the aim of initiating proper treatment and/or follow-up at the earliest [1].

## Data Availability

All the data supporting our findings is contained within the manuscript.

## Abbreviations

CAN: Crystalglobulin-associated nephropathy
MGRS: Monoclonal gammopathy of renal significance
MBL: Monoclonal B-cell lymphocytosis
MM: Multiple myeloma
MGUS: Monoclonal gammopathy of undetermined significance
CLL: Chronic lymphocytic leukemia
IEM: Immunoelectron microscopy
